# Selections

**Published:** 1816-05

**Authors:** 


### PART III.

### SELECTIONS.

### FOREIGN JOURNALS.

#### *Memoir on a Case of poisoning by Opium.*†

"It frequently happens, that the relations which exist between the symptoms determined by a poisonous substance, and the organic lesions consequent on its action, cannot be recognized. Sometimes a substance of this kind is found in the organs after death, without its presence having been at all suspected in the living subject. Notwithstanding the facility with which experiments are made upon animals, we can never certainly conclude that any lesion is produced by a poison, unless it be discovered on the morbid organ, a mean of proof belonging to the domain of chemistry. This science, considered as a branch of forensic me-

---

† By Edward Petit, M. D. and M. P. H. Petit, of Corbeil. See *Bulletin de la Société Medicale d'Emulation, Novembre* 1815. We have selected this Memoir not merely from the value of the facts which it contains, but as an example of the diligence and precision exercised by our neighbours in medico-legal researches—an example which we earnestly recommend to the notice and imitation of practitioners on this side the channel.—Editors.

dicine, is susceptible of great improvement. We may indeed imitate all the phænomena displayed in the poisoning of a human subject; but can the high perfection, with which our experiments on mineral poisons are executed, be attained in our operations upon vegetable substances?

"The following fact and experiments are published as useful contributions to this subject:

"M. C. a miniature painter, had studied pharmacy. He was aged 38. On the 19th of June, 1814, he called at an inn which he occasionally frequented. It was late. He complained of fatigue, and desired to have a bed and some lemonade. The inn-keeper observed that his dress was disordered; and took him, when lying down, a glass of lemonade. He seemed to suffer greatly, but would not send for a physician. He asked a girl to obtain for him some white vitriol, which she could not do. A female acquaintance saw him for a moment. It is supposed that the vitriol was procured by her, as he had certainly employed some.

"About four o'clock in the morning, a servant, hearing a noise in the sick man's chamber, inquired if he wanted any thing: but, finding him senseless, awakened the inn-keeper, who sent for Dr. Edward Petit. The latter saw him at six o'clock, presenting the following symptoms: Face pale; features nearly natural; eye-lids closed; eyes fixed, and turned upwards; respiration moaning; palpitations very remarkable, but regular; skin hot; pulse frequent, but regular. A draught of musk and sulphuric æther could not be got down.

"Blisters were applied to the legs. Four hours afterwards, respiration became short and fitful; the features sunk; the skin cold. Death shortly ensued.

"During his absence, a pen-knife, with many blades, had been discovered in the chamber of the deceased; and one of them was loaded with a vegetable extract, apparently opium. About a decigram * of it was collected. A piece of brown paper was also found, which had a narcotic smell, and seemed to have enclosed a similar substance; and a glass on the chimney-piece was half full of turbid wine, apparently holding a foreign body in solution.

"The magistrates, informed of this event, directed the body to be examined; although, beneath the pillow of the deceased, a letter was found, announcing the purpose of self-destruction. Dr. Petit was charged with the examination, and we proceeded conjointly to it.

"Arrived at the chamber of the deceased, we secured, 1st, the pen-knife, covered with extractive matter; 2dly the paper, yet containing a small portion; and 3dly, we poured the remnant of the wine into a phial, which was corked, sealed, and ticketed No. 1.

"Examination of the body presented the following appear-

* Two grains.—Edit.

ances: — *External.* Features natural; evacuation by the mouth of a brown frothy liquid; ecchymosis of the integuments of the neck, and superior and lateral parts of the trunk; slight vesicles.

" *Abdomen.* Exhalation, upon opening it, of a vinous odour; flesh of a deep red colour; slight redness upon some points of the small intestine; inferior surface of the liver of a green black colour to a considerable extent. Great quantity of air contained in the stomach, and the smell of opium very remarkable. About four ounces of a brownish liquid were collected, and put into a phial corked, sealed, and marked No. 2. The internal surface of the stomach was covered by a sort of brownish mucous layer, and unequally inflamed at different points, but more strongly at its superior part. The intestinal mucous membrane was in the same state, and apparently affected with chronic catarrh. The small intestine contained only gas; the large one, a little fæcal matter. The internal surface of the latter was very pale, and the gas extricated from it had a vinous odour.

" *Thorax.* Slight and old adhesions on the left side; lungs gorged with blood; the heart flaccid, and both its ventricles containing coagulated blood; brownish froth found in the larynx; the œsophagus sound; its internal surface, and that of the mouth, pale-coloured.

" *Cranium.* Vessels of the hairy scalp, sinuses and vessels of the brain, gorged with blood; the cerebral mass voluminous; little serum contained in its ventricles.

" From this examination, and the letter of the deceased, Dr. Petit thought fit to make the following declaration, which was attached to the *process-verbal* of the magistrate: '*That, considering the symptoms remarked twice yesterday, and the dissection of the body performed this day, he thought this man had died from the effect of a narcotic poison, which he presumed to be opium;*' and, in order to acquire greater certainty respecting the cause of this event, M. Petit, apothecary, instituted the following analysis and experiments.

" *Analysis of the liquid found in the chamber of the deceased, and ticketed No.* 1. This liquid, when filtered, was transparent, of a bright red colour; weighed six drachms; left on the filter a very light brown precipitate, but too minute to be submitted to experiment; had a vinous odour and styptic taste.—The following tests gave for result: — *The water and muriate of barytes,* a liquid less coloured, and an abundant white rose precipitate. *Hydro-sulphuret of potash,* an almost colourless liquid, and a slate-coloured precipitate. *Liquid ammonia,* a yellowish liquid, with a radiated pellicle on its surface, and abundant curdled precipitate of the colour of wine-lees, not dissolved by excess of ammonia. *Carbonate of potash,* a liquid of a light violet hue; radiated pellicle on the surface; abundant curdled precipitate of a violet black. *Prussiate of potash,* abundant curdled precipitate; some appearances of iron.

" These tests demonstrate the presence of a sulphate, presumed to be that of zinc, on account of the solubility of the precipitate by ammonia, in an excess of this alkali. M. Petit treated four drachms of this liquid with *pure potash.* The precipitate, washed and dried, he mixed with powdered charcoal, and obtained from it, by exposure to heat, in a glass tube closed at one extremity, a metallic particle, apparently zinc.

" *Analysis of the liquid found in the stomach, and preserved in the phial No.* 2. This liquid was of a fawn grey colour, turbid, flaky; containing green peas in suspension; weighing about four ounces; of a nauseous fœtid smell, much resembling that of crude opium.

" *Action of tests upon the filtered liquid.*—*Litmus paper* reddened. *Turmeric* changed to a canary yellow. *Infusion of violets* reddened. *Litmus paper*, which had been *reddened* by an acid, unchanged. *Water and muriate of barytes* rendered slightly turbid. *Lime-water* precipitated white. *Alcohol*, dirty white, flaky, copious precipitate. *Ammonia*, a light whitish cloud. *Carbonate of potash*, deepened the colour of the liquid. *Sulphate of copper*, a green bluish precipitate. *Hydro-sulphuret of potash*, a white precipitate. *Prussiate of potash*, no change. *Acetate of lead*, white precipitate soluble in nitric acid. *Sulphuric acid*, liquid slightly turbid; acetic acid disengaged. *Sulphurous acid*, yellowish precipitate, assuming, after six hours, a deeper colour. *Nitric acid*, a yellowish white precipitate. *Muriatic acid*, the same. A drop poured upon a *plate of silver*, and exposed to the sun, left a very elastic gelatinous substance, which produced no change on the plate by friction. A *plate of copper* oxydized green.

" These trials demonstrate the presence of the acetic and carbonic acids; and were made in the intention of discovering if the liquid contained other poisonous substances besides opium.

" One ounce of the liquid, passed through the filter, left upon it two decigrams, five centigrams,* of a grey matter, which burnt like an animal substance. Evaporated in a sand-bath, it gave out an acid fœtid smell, in which that of opium was clearly distinguished: it became turbid in concentrating, and took the consistence of a semi-transparent jelly, clear brown, and weighing two grams. Of this gelatinous substance, treated by alcohol until the latter came off colourless, there remained six grams, three decigrams,† of a tenacious matter, corrugated by a fresh addition of alcohol. A small quantity, thrown upon burning coals, exhaled the smell of burnt horn, and then of bread-crumb. It dissolved in distilled water, which put on a milky appearance, and had a fœtid smell. The alcoholic solutions combined had a deep amber colour; the smell of opium, although somewhat less nauseous; and a salt bitter taste. During evaporation, it remained transparent; and formed, on the surface, small star-like crystals. Evaporated to the consistence of an extract, it attracted the mois-

* About five grains.—Edit.

† This we cannot comprehend: it is evidently a blunder.—Edit.

ture of the air, and had a smell of opium less decided. This extract dissolved in distilled water with the exception of five centigrams of a brownish matter. The solution reddened *litmus paper*, and was precipitated by *nitrate of silver*. Set to evaporate, I removed with care the small crystals which formed on the surface of the liquid. They weighed altogether fifteen centigrams. One drop of sulphuric acid extricated the smell of muriatic acid. The liquid, evaporated to the consistence of extract, weighed one gram.

"Thus we thought that we had obtained the proof of the presence of an acid, indicated by the smell exhaled during evaporation, and by the action of the different tests:—of an animal matter combined with mucilage, shewn by the smell resulting from its combustion, and by the nature of the residue, difficult of incineration;—of a muriate, presumed to be that of soda, from the deliquescence of the salt obtained, the smell of muriatic acid disengaged on the addition of sulphuric, and the precipitate of nitrate of silver;—of an extract, believed to be that of opium, from the smell of the evaporations, and its bitter taste. Having performed the last analysis upon somewhat less than a fourth of the fluid collected (and this was not all which the stomach contained) we may calculate by approximation, that the deceased had swallowed at least a drachm of the vegetable extract.

"*Employment of the extract obtained by concentration of the contents of the stomach.* A small river hog,* ten days old, having fasted ten hours, swallowed, by force, fifteen centigrams of the extract in four grams of distilled water. Soon after ingestion, stiffness of the anterior limbs; general convulsions; death in three minutes. In order to introduce the liquid, it was necessary, several times, to compress the animal's nostrils. On examination, thirty-six hours after death, the lungs were found of a bright red colour, containing little blood; the left ventricle of the heart contracted; auricles black; stomach readily lacerable; flesh of a deep red colour; the blood still fluid.

"Another river hog, somewhat more lively than the former, having just taken food, was forced to swallow a centigram of the extract, in four grams of water; but, the precaution of securing the nostrils having been neglected, three-fourths of the dose were spilled. After ten minutes, the animal was affected with universal trembling; and then became tranquil. Every symptom had disappeared in twenty minutes.

"After five hours, two centigrams of the extract were given to the same animal. In ten minutes, the anterior extremities became stiff; then the posterior. Difficulty of progression, an hour and a half afterwards. The animal moaned, the head drooped, and was again suddenly raised. In the evening, it appeared more lively; but, on the morning after, was found dead, stiff, and swollen. An accident prevented examination of the body.

---

* *Cabiai*, an amphibious animal, a native of America. Edit.

" The difference of operation of the same substance on these two subjects is readily explained: the one had fasted; the other been recently fed. About fifteen centigrams of extract were given to the former; at first, five centigrams, and then double that dose, five hours afterwards, to the latter.*

" We could collect from the pen-knife and brown paper only five centigrams of an extract which had the smell, taste, and colour of extract of opium. It was mixed with four grams of water, and given to a lively river hog, seven days old. An hour afterwards, it could no longer run: two hours afterwards, it fell upon its hind quarters: the left eye wept, was half closed, and appeared paralysed. Four hours afterwards, horripilation; it fell frequently, and no longer took food. Six hours afterwards, it was benumbed. On the following morning, we found it dead. No signs of alteration in any of the organs; blood fluid and blackish.

" In witnessing melancholy events, such as that now related, we cannot but regret that the pharmaceutical police displays such want of vigour and activity; and that, under the name of druggists, a crowd of ignorant persons dispense medicines, with the properties of which they are unacquainted. It were also much to be wished, that apothecaries, impressed with a sense of their duty, would not sell powerful drugs, except by the direction of the physician. Without this precaution, they may frequently become the involuntary instruments of such accidents as the following, which recently occurred.

" An English officer, bent on self destruction, purchased two ounces of liquid laudanum, under a false pretext. He swallowed it, having just before taken coffee with milk. Shortly afterwards, he vomited much. For some hours, he continued in a state of stupor, followed by a restless melancholy, with recurrence of the propensity to suicide. Yet he was better. From Paris, he returned to England. Thus this officer resisted the influence of a dose of extract of opium (by calculation about a drachm) which had sufficed to destroy the subject of the first case. To the nature of the preparation of the drug, and difference of position of the individuals, this variety of result may be attributed.

" We think that, from these facts and experiments, nothing can be inferred respecting the action of sulphate of zinc, which the patient had probably taken with a view of counteracting the operation of the opium; since, it is most likely, that he had swallowed this substance when the sensibility and contractility of the stomach were already abolished; and we consider it certain, that M. C. poisoned himself with a large dose of extract of opium, which produced no other organic lesion than such as may result from inflammation of the mucous membrane of the stomach."

---

* This is a direct and palpable contradiction. The present statement we believe to be the true one; and that, in the former, *grains* should have been written instead of *centigrams*.—Edit.

This Memoir is terminated by the annunciation of what the writers please to designate a *new* and important fact in the medical history of opium. The accuracy and importance are alike unquestionable: its *novelty* we are strongly disposed to contest; for we believe, that there are few medical men of observation and experience, unacquainted with the property which opium possesses, and frequently exercises, of inducing *furred tongue, bitterness of the mouth, and all the phænomena of gastric derangement,* or *gastricity,* as it is here termed. Would that we could impress upon the recollection of Doctor and Monsieur Petit a truth certainly quite novel to themselves, and of no little importance to all writers on medicine. Description rigorous and unvarying, and a lucid style of narration, are little less essential to the productions of science, than the accuracy and value of the materials of which they are composed! In this respect, the present memoir is singularly unfortunate.—Editors.

---

